# *CRISPR/Cas9* Deletion of *SOX2 Regulatory Region 2* (*SRR2*) Decreases SOX2 Malignant Activity in Glioblastoma

**DOI:** 10.3390/cancers13071574

**Published:** 2021-03-29

**Authors:** Ander Saenz-Antoñanzas, Veronica Moncho-Amor, Jaione Auzmendi-Iriarte, Alejandro Elua-Pinin, Karine Rizzoti, Robin Lovell-Badge, Ander Matheu

**Affiliations:** 1 Cellular Oncology Group, Biodonostia Health Research Institute, 20014 San Sebastian, Spain; ander.saenz@biodonostia.org (A.S.-A.); Jaione.auzmendi@biodonostia.org (J.A.-I.); alejandro.elua.pinin@gmail.com (A.E.-P.); 2 Stem Cell Biology and Developmental Genetics Lab, The Francis Crick Institute, London NW1 1AT, UK; veronica.moncho@biodonostia.org (V.M.-A.); Karine.Rizzoti@crick.ac.uk (K.R.); Robin.Lovell-Badge@crick.ac.uk (R.L.-B.); 3 Donostia Hospital, 20014 San Sebastian, Spain; 4 CIBER of Frailty and Healthy Aging (CIBERfes), Carlos III Institute, 28029 Madrid, Spain; 5 IKERBASQUE, Basque Foundation for Science, 48009 Bilbao, Spain

**Keywords:** SOX2, SRR2, glioblastomas, signaling, cancer stem cells

## Abstract

**Simple Summary:**

Understanding how SOX2, a major driver of cancer stem cells, is regulated in cancer cells is relevant to tackle tumorigenesis. In this study, we deleted the *SRR2* regulatory region in glioblastoma cells. Our data confirm that the *SRR2* enhancer regulates SOX2 expression in cancer and reveal that *SRR2* deletion halts malignant activity of SOX2.

**Abstract:**

SOX2 is a transcription factor associated with stem cell activity in several tissues. In cancer, SOX2 expression is increased in samples from several malignancies, including glioblastoma, and high SOX2 levels are associated with the population of tumor-initiating cells and with poor patient outcome. Therefore, understanding how SOX2 is regulated in cancer cells is relevant to tackle tumorigenesis. The *SOX2 regulatory region 2* *(SRR2)* is located downstream of the *SOX2* coding region and mediates *SOX2* expression in embryonic and adult stem cells. In this study, we deleted *SRR2* using *CRISPR/Cas9* in glioblastoma cells. Importantly, *SRR2*-deleted glioblastoma cells presented reduced SOX2 expression and decreased proliferative activity and self-renewal capacity in vitro. In line with these results, *SRR2*-deleted glioblastoma cells displayed decreased tumor initiation and growth in vivo. These effects correlated with an elevation of p21^CIP1^ cell cycle and p27^KIP1^ quiescence regulators. In conclusion, our data reveal that *SRR2* deletion halts malignant activity of SOX2 and confirms that the *SRR2* enhancer regulates SOX2 expression in cancer.

## 1. Introduction

SOX2 is a member of the SOX family of high mobility group (HMG) box transcription factors, and plays an important role in the maintenance of embryonic stem cell pluripotency and progenitor identity during embryogenesis and in adulthood [1]. In the embryonic central nervous system, SOX2 is expressed throughout the neural tube during early stages of neurogenesis and in progenitors as development progresses. It is also required for neural stem cell (NSC) activity in the adult. Several studies describe how SOX2 expression is elevated in a large proportion of cancers, including brain tumors such as glioblastoma (GBM) [2,3,4,5,6].

GBM is the most common malignant primary brain tumor in adults. The average survival of patients diagnosed with GBM is around 15 months [7]. GBMs are notorious for their resistance to therapy, which has been linked to genetic and cellular heterogeneity [8]. Among these, the glioma stem cell (GSC) subpopulation displays stem cell characteristics such as unlimited self-renewal capacity, quiescence and multilineage differentiation potential, being responsible for tumor initiation, progression, therapy resistance and tumor recurrence [9]. Several studies reported overexpression of SOX2 in GSC subpopulations and in patient samples, and the high levels are associated with tumor aggressiveness and worse prognosis. Furthermore, downregulation of *SOX2* in GSCs impairs proliferation, self-renewal and their ability to form tumors in vivo, whereas *SOX2* overexpression promotes cell proliferation, self-renewal capacity and tumorigenesis [3,10,11,12,13,14,15]. These results show SOX2 as a relevant driver in GBM progression.

Transcriptional regulation of *SOX2* is complex and not fully understood. The expression of the *SOX2* gene in embryonic stem cells (ESCs) and multipotent neural progenitor cells in the ventricular zone of embryonic brains is supported in major part by a set of regulatory regions: *SRR1*, *SRR2*, *SRR18*, *SRR107* and *SRR111* [16,17,18]. In particular, *SRR2* is located ~4 kb downstream of the *SOX2* coding region and has a regulatory core sequence comprising octamer and *SOX2* binding sites. In ESCs and NSCs, *SRR2* mediates *SOX2* activation by recruiting OCT3-4-SOX2 or OCT6-SOX2, respectively, and, on their differentiation, *SRR2* recruits p130-E2F4-SIN3A repressive complexes [17,19]. This is in part mediated by the association of p21^CIP1^ and p27^KIP1^ to *SRR2*. Indeed, when p27^KIP1^ binds to *SRR2*, together with the p130-E2F4-SIN3A complex, this contributes to the repression of *SOX2* and leads to differentiation programs in ESCs, mouse embryonic fibroblasts (MEFs) and teratoma cells [20]. Similarly, p21^CIP1^ controls the proliferation of NSCs in the adult brain via *SRR2* binding [21]. In breast cancer, high transcriptional activation of *SOX2*, monitored by an *SRR2* reporter, has been associated with higher tumorigenic capacity in a small subset of cancer stem cell phenotypes (CD44+/CD24-) in triple-negative breast cancer cell lines and patient samples, showing the potential relevance of *SRR2* in tumorigenesis [22,23].

Thus, in the present study, we used *CRISPR/Cas9* technology to delete *SRR2* in GBM cells. We observed a reduction of SOX2 expression, which is accompanied by impairment of proliferative activity and reduction of self-renewal capacity in vitro. Additionally, *SRR2*-deleted cells lose the capacity to form tumors in vivo. These effects correlated with an elevation of the p21^CIP1^ and p27^KIP1^ cell cycle and quiescence regulators. These results show that *SRR2* is required for SOX2-driven oncogenic activity in GBM cells.

## 2. Materials and Methods

### 2.1. Single Guide RNA (sgRNA) Design for SRR2 Enhancer Deletion

Single guide RNAs (sgRNAs) were designed (www.crispr.mit.edu (accessed on 22 January 2021)) to flank and delete the human *SRR2* enhancer (200 bp), sited ~4 kb downstream of the *SOX2* gene (UCSC hg19 genome chr3: 181,429,712–181,435,223) by *CRISPR/Cas9* genome editing. Two guides were designed for targeting the 5′ side of *SRR2* (G7_5′ and G5_5′) and two guides for the 3′ side of *SRR2* (G1_3′ + G3_3′) (Table S1), while putative off-targets were determined using the MIT CRISPR Design tool (http://crispr.mit.edu/ (accessed on 22 January 2021), which indicated top scoring off-target sites. sgRNAs and Cas9 mRNA were prepared as previously described [24]. U373MG cells were transfected with plasmid Cas9 (*pSpCas9(BB)-2A-Puro* (PX459) V2.0, PX459, #62988, Addgene) including four sgRNAs or only Cas9 empty vector (named control), following previously described protocols [24]. Forty-eight hours after transfection, cells were selected using 2 μg/mL puromycin. Single, puromycin-resistant cells were selected and expanded for genomic DNA extraction. In particular, 2 puromycin-resistant clones were isolated with cloning rings in the *pSpCas9(BB)-2A-Puro* (PX459) and four sgRNA plate (U373MG *SRR2^del^* clone2 and U373MG *SRR2^del^* clone5). Thus, we generated three different cell lines: control (only transfected with plasmid PX459), U373MG *SRR2^del^* clone2 (deletion of *SRR2*) and U373MG *SRR2^del^* clone5 (deletion of *SRR2*). Additionally, U373MG parental cells were also used in the experiments to check whether Cas9 empty vector (control) had any effect on U373MG cells.

### 2.2. Genotyping SRR2 Enhancer and Sequencing of Clones 2 and 5

Genomic DNA was extracted from U373MG cells and deleted *SRR2* regulatory region and clones and control were identified by PCR genotyping (Table S2). The primer pair *SRR2*-F and *SRR2*-R amplified a 724 bp product in wild type cells and a product of around 200-400 bp cells carrying the *SRR2* deletion when we used all guides. Sequencing revealed a difference of 1 nucleotide between both clones; clone2 deletion was 479 bp whilst clone5 deletion was 480 bp. Detailed information of sequencing of clones is presented in Figure S1. Sequencing was carried out in a 3130xl Genetic Analyzer (Applied Biosystems, Carlsbad, CA, USA)).

### 2.3. Chromatin Immunoprecipitation

Cells were crosslinked with 1% formaldehyde for 15 min at room temperature. Crosslinking was stopped by the addition of glycine to a final concentration of 0.125 M. Fixed cells were lysed in lysis buffer (1% SDS, 10 mM EDTA, 50 mM Tris-HCl, pH 8.0) and sonicated. An aliquot of 60 μg was reserved as input. For immunoprecipitation, 600 μg of protein were diluted in dilution buffer (1% Triton X-100, 2 mM EDTA, 150 mM NaCl and 20 mM Tris-HCl, pH 8.0, containing protease inhibitors), and pre-cleared with A/G plus-agarose (SantaCruz). The antibodies used for the immunoprecipitation were p27^KIP1^ (Santa Cruz, C-19) and IgG (Santa Cruz, SC-2020). Immune complexes were precipitated with A/G plus-agarose and washed sequentially with low-salt immune complex wash buffer (0.1% SDS, 1% Triton X-100, 2 mM EDTA, 20 mM Tris-HCl, pH 8.1, 150 mM NaCl), high-salt immune complex wash buffer (0.1% SDS, 1% Triton X-100, 2 mM EDTA, 20 mM Tris-HCl, pH 8.1, 500 mM NaCl), LiCl immune complex wash buffer (0.25 M LiCl, 1% NP-40, 1% deoxycholate-Na, 1 mM EDTA, 10 mM Tris-HCl, pH 8.1) and TE buffer, and finally eluted in elution buffer (1% SDS, 0.1 M NaHCO3). All samples, including inputs, were de-crosslinked, treated with proteinase K and DNA was extracted with phenol–chloroform and resuspended in TE buffer. All PCR reactions were carried out in triplicate. Primers used for PCR after Chromatin Immunoprecipitation (ChIP) are shown in Table S2.

### 2.4. Cell Lines and Cultures

A172, T98G, U87MG, U251MG and U373MG were purchased from the ATCC and cultured in Dulbecco’s Modified Eagle Medium (DMEM, Gibco, Waltham, MA, USA, supplemented with 10% Fetal Bovine Serum (FBS, Gibco, St. Louis, MO, USA), 100 U/mL penicillin and 100 µg/mL streptomycin, for monolayer cultures. For oncosphere studies, U373MG cells were cultured in Glioblastoma Stem Cell GSC medium consisting of DMEM/F12 (Sigma, Waltham, MA, USA) supplemented with N2, B27 (Fisher,Waltham, MA, USA) and growth factors: 20 ng/mL basic fibroblast growth factor (bFGF) and 20 ng/mL epidermal growth factor (EGF) (Sigma, Waltham, MA, USA). Patient-derived GNS166 and GNS179 stem cell lines, kindly provided by Dr. Steven Pollard, and GB1012 and GB1034, established by our group [15], were cultured in GSC medium. All cells were maintained at standard conditions of 37 °C and 5% CO_2_ in a humidified atmosphere.

Neurosphere isolation was performed as previously described [25] with some modifications. Briefly, animals were killed by raising the concentration of CO_2_, and the brain was put into Phosphate-Buffered Saline (PBS) 1X (Gibco, St. Louis, MO, USA). The subventricular zone (SVZ) was isolated and centrifuged at 230× *g* for 5 min. After removing the supernatant, the tissue was mechanically disrupted by pipetting up and down and enzymatically disaggregated using 5 mL DMEM/F12 with 10% papain (Roche), previously activated for 30 min at 37 °C and with 25 U of DNase (Promega, Madison, Wisconsin, USA). Cells were maintained at 37 °C for 30 min in rotation (180 rpm), before being centrifuged at 230× *g* for 5 min. After centrifugation, cells were washed with PBS and re-suspended in 2 mL of complete DMEM/F12 supplemented with 40 ng/mL Epidermal Growth Factor (EGF) and 40 ng/mL basic Fibroblast Growth Factor (bFGF). The culture was grown as spheres in suspension and the medium was renewed twice a week. For differentiation assays, 50 neurospheres were seeded in chamber slides (Lab-Tek II Chambers, 1023-4121, Thermo Scientific, Budapest, Hungary) pretreated with 10 µg/mL laminin (L2020, Sigma-Aldrich, St. Louis, MO, USA) at 37 °C for 3 h. Cells were maintained in culture for 7 days in complete DMEM/F12 without EGF and bFGF. Half of the medium was replaced every two days.

### 2.5. Lentiviral Infections

For stable *SOX2* overexpression, lentiviral infections were performed as previously described [15]. We used a *pLM-mCitrine-SOX2* construct (SOX2) and a *pWPXL-GFP* construct (GFP) as controls. Cells were infected at a multiplicity of infection of 10 for 6 h.

### 2.6. Immunofluorescence

Immunofluorescence was performed as described in previous studies [26]. Cells were incubated with SOX2 (AB5603 Millipore, Burlington, MA, USA) and phospho-histone3 (pH3) (ab14955 Abcam, Cambridge, UK) antibodies. Secondary antibodies of anti-mouse Alexa Fluor 555 IgG (Invitrogen, Carlsbad, CA, USA) and anti-rabbit Alexa Fluor 488 IgG (Invitrogen, Carlsbad, CA, USA) were used. Nuclear DNA was stained with Hoechst 33342 (Sigma, St. Louis, MO, USA). Pictures were taken with an Eclipse 80i microscope and processed with the NIS Elements Advances Research software (Nikon, Tokyo, Japan).

### 2.7. Western Blot Analysis

Immunoblots were performed following standard procedures [26]. Specific antibodies against SOX2 (AB5603 Millipore, Burlington, MA, USA), p21^CIP1^ (sc-397 Santa Cruz, Dallas, TX, USA), p27^KIP1^ (sc-1641 Santa Cruz, Dallas, TX, USA), BMI1 (05-637 Millipore, Burlington, MA, USA) and β-actin (A5441, Sigma, St. Louis, MO, USA) were used in the study. For secondary antibodies, horseradish peroxidase (HRP)-linked anti-rabbit (7074S Cell Signaling, Danvers, MA, USA), anti-mouse (7076S Cell Signaling, Danvers, MA, USA) or anti-goat (sc-2020 Santa Cruz, Dallas, TX, USA) were used. Detection was performed by chemiluminescence using NOVEX ECL Chemi Substrate (Thermo Fisher, Waltham, MA, USA).

### 2.8. RNA Analysis

Total RNA extraction was performed by Trizol (Life Technologies, Budapest, Hungary). Reverse transcription was performed using random priming and Maxima First Strand cDNA Synthesis Kit (Thermo Fisher, Waltham, MA, USA), according to the manufacturer’s guidelines. To analyze gene expression, quantitative real-time polymerase chain reaction (qRT-PCR) with 20 ng of cDNA was performed by Absolute SYBR Green mix (Thermo Scientific, Budapest, Hungary) in a LightCycler 96 thermo-cycler (BioRad, Hercules, CA, USA). Transcript levels were normalized to *GAPDH* and measured using the ΔΔCt relative quantification method.

### 2.9. Proliferation and Colony Formation Assays

For cell count experiments, a total of 2.5 × 10^4^ cells per well were seeded in duplicate in 6-well treated plates. The total number of cells for each condition was determined 1, 3 and 5 days after culture using a Neubauer counting chamber and a light microscope. For colony formation experiments, a total of 500 cells per well were seeded in duplicate in 6-well treated plates. After 10 days, cells were fixed with 4% paraformaldehyde for 30 min and stained with Giemsa (Sigma, St. Louis, MO, USA) 5% in PBS for another 30 min.

### 2.10. Oncosphere Formation Assay

For the oncosphere formation assay, 10,000 cells/well were seeded in non-treated 6-well flat-bottom plates. Primary oncospheres (1^ry^ GSCs) were grown for 7–10 days in GSC medium, and after quantification, spheres were mechanically and enzymatically disaggregated with accutase (Gibco, Waltham, MA, USA). Then, they were seeded for secondary oncosphere (2^ry^ GSCs) formation and maintained for another 7–10 days. Fresh medium was added every 2–3 days to the plate.

### 2.11. In Vivo Carcinogenesis Assay

All animal handling and protocols were approved by the animal care ethic committee of the Biodonostia Institute (PRO-AE-SS-100). For subcutaneous xenografts, U373MG *SRR2^del^* control, clone2 and clone5 cells were harvested with trypsin/EDTA and resuspended in PBS before injection. A tumor initiation assay was performed by injecting 5 × 10^5^ and 5 × 10^4^ cells into both flanks of 8-week-old Foxn1^nu^/Foxn1^nu^ nude mice. In the assay, external calipers were used to measure tumor size twice a week. From these measurements, tumor volume was estimated by V = L × W^2^ × 0.5; where L is tumor length and W is the tumor width.

### 2.12. Immunohistochemistry (IHC)

Tumors generated in mice were dissected, fixed in 4% formalin for 48 h and embedded in paraffin. Four-micrometer thick sections were deparaffinized, rehydrated and heated for 10 min in citrate buffer for antigen retrieval. Endogenous peroxidase was blocked with 5% hydrogen peroxide in methanol for 15 min. After incubation with blocking solution, sections were incubated with the respective primary antibody, anti-SOX2 (ab97959 Abcam, Cambridge, UK), anti-Ki67 (ab15580, Abcam, Cambridge, UK), anti-p21^CIP1^ (sc-397 Santa Cruz, Dallas, TX, USA) and anti-p27^KIP1^ (sc-1641 Santa Cruz, Dallas, TX, USA), at 37 °C for 2 h. The sections were then washed and incubated with MACH 3 Rabbit Probe and MACH 3 Rabbit HRP-Polymer (M3R531, Biocare Medical, Pacheco, CA, USA). Color was developed with 3,3′-Diaminobenzidine (DAB) and nuclei were counterstained with hematoxylin.

### 2.13. Statistical Analysis

All measurements were represented as mean values ± standard error (SEM) and analyzed by Prism v.8.0c (GraphPad Software, San Diego, CA, USA). Data between two groups were compared by an unpaired *t*-test, while those among multiple groups were tested using one-way analysis of variance (ANOVA), followed by Tukey’s post hoc test. For Extreme Limiting Dilution analysis (ELDA), we used a chi-square test (χ^2^). Standard significance levels were used: * *p* ≤ 0.05; ** *p* ≤ 0.01; and *** *p* ≤ 0.001.

## 3. Results

### 3.1. Characterization of SOX2 Expression and Activation of SRR2 Enhancer in Glioma Cells

**Figure 1:**
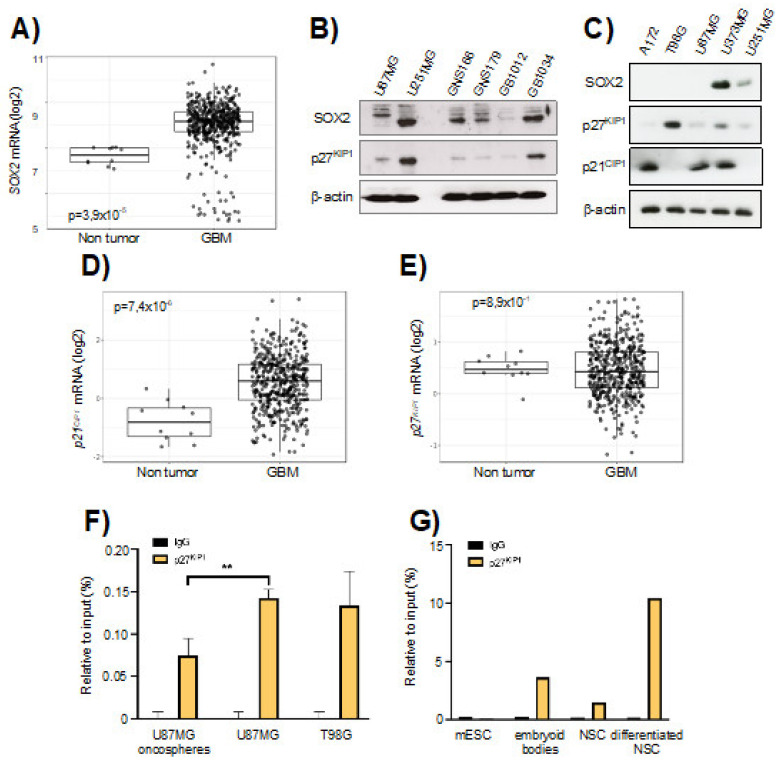
*SOX2-SRR2* in glioma cells. (**A**) *SOX2* mRNA expression in control (*n* = 10) and GBM samples (*n* = 528) from The Cancer Genome Atlas (TCGA) cohort in GlioVis database (http://gliovis.bioinfo.cnio.es (accessed on 22 January 2021)). (**B**) Immunoblotting of SOX2 and p27^KIP1^ in U87MG and U251MG cell lines, in GNS166 and GNS179 patient-derived stem cell lines and in GB1012 and GB1034 GBM biopsies, β-actin was used as loading control. (**C**) Immunoblotting of SOX2, p27^KIP1^ and p21^CIP1^ in A172, T98G, U87MG, U373MG and U251MG cells, β-actin was used as loading control. (**D**) p21^CIP1^ and (**E**) p27^KIP1^ mRNA expression in control (*n* = 10) and glioblastoma (GBM) samples (*n* = 528) from The Cancer Genome Atlas (TCGA) cohort in GlioVis database. (**F**) Chromatin immunoprecipitation (ChIP) of p27^KIP1^ at *SOX2-SRR2* of two different stages: U87MG oncospheres *vs*. U87MG parental cells and T98G parental cells (*n* = 3). The statistical significance was assessed by ANOVA test: * *p* < 0.05, ** *p* < 0.01 and *** *p* < 0.001. (**G**) ChIP of p27^KIP1^ in the *SOX2-SRR2* of mouse embryonic stem cells (mESCs) and neurospheres (neural stem cells, NSCs) before and after differentiation (*n* = 2).

We analyzed the expression of SOX2 in the TCGA cohort of human GBM samples and compared them with healthy brain tissue. The expression of *SOX2* was upregulated in GBM samples (Figure 1A). Similarly, Western blotting revealed very high expression of SOX2 in freshly derived glioma stem cell (GSC) cultures from human patients and in U251MG and U373MG cell lines, while A172, T98G and U87MG expressed low levels (Figure 1B,C). The levels in U251MG and U373MG cells are within the range of expression observed in tumor biopsies [15], suggesting that those levels are of biological relevance.

Since the association of p21^CIP1^ and p27^KIP1^ partly mediates the effect of *SRR2* on SOX2 repression in several cell types [20], we studied the expression of p21^CIP1^ and p27^KIP1^ in glioma cell lines and biopsies. The levels of *p21^CIP1^* were elevated in GBM from the TCGA cohort, whilst *p27^KIP1^* ones were slightly reduced (Figure 1D,E). The levels of p27^KIP1^ were also generally low in GSCs and glioma cells lines (Figure 1B,C). Glioma cell lines cultured under stem cell media grow as oncospheres, which express a striking elevation of SOX2 levels [15]. To characterize the impact of *SRR2* enhancer in SOX2 expression on glioma cells, we performed ChIP assays in U87MG and T98G parental and U87MG oncosphere populations, studying the presence or absence of p27^KIP1^ in this region. Interestingly, ChIP assays revealed that p27^KIP1^ is highly enriched in *SRR2* in U87MG and T98G parental cells and significantly reduced in U87MG oncosphere subpopulations (Figure 1F). We also studied the presence or absence of p27^KIP1^ in *SRR2*, in both normal and differentiated NSCs. For this, neurospheres generated in vitro from freshly dissected brains were used. Differentiated neurospheres showed an enrichment of p27^KIP1^ on *SRR2* compared to non-differentiated neurospheres (Figure 1G). p27^KIP1^ was recruited to *SRR2* in differentiated mouse embryonic stem cells (mESCs) cultured as embryoid bodies (Figure 1G), corroborating previous studies [20]. Altogether, these results suggest that *SRR2* is involved in SOX2 activation in stem cells in physiological and pathological conditions in the brain, at least in part through p27^KIP1^ binding.

### 3.2. SRR2 Regulates SOX2 Expression in GBM Cells

**Figure 2:**
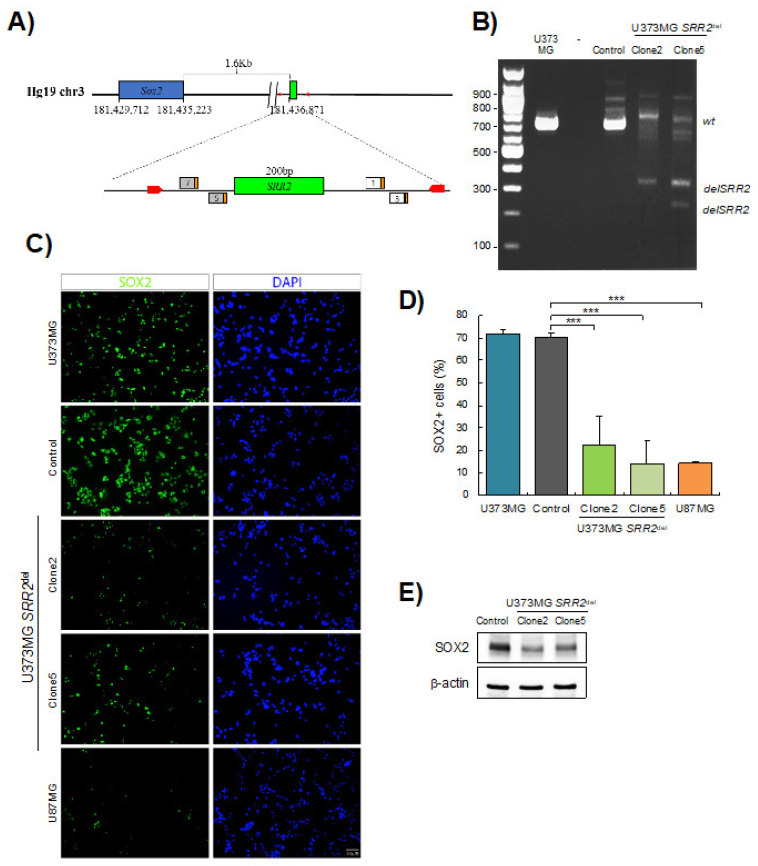
SOX2 levels are reduced in *SRR2*-deleted cells. (**A**) Schematic representation of *SOX2* and *SRR2* downstream. At the bottom, magnification of the *SRR2* surrounding region. *SRR2* (200 bp) is marked by a green box. The location of the guides in 5′ (guide 7 and guide 5) and in 3′ (guide 1 and guide 3) single guide RNA (sgRNA) is labeled including their protospacer adjacent motif (PAM) sequence (orange box). Primers used for genotyping the deletion are marked with red arrows. (**B**) Gel electrophoresis showing loss of *SRR2* in U373MG *SRR2^del^* clone2 and clone5 vs. control or parental cell line U373MG after transfection with guides and Cas9. (**C**) Representative images of SOX2 immunofluorescence (scale bar = 50 μm) and (**D**) quantification of SOX2-positive cells in U373MG (parental cells), control cells, U373MG *SRR2^del^* clone2 and clone5 and U87MG cells (*n* = 3). (**E**) Representative immunoblot of SOX2 in control, clone2 and clone5 cells. The statistical significance was obtained with the Student’s *t*-test (*** *p* < 0.001).

Next, we engineered a deletion of *SRR2* using *CRISPR/Cas9* technology in U373MG cells (Figure 2A), which express high SOX2 levels (Figure 1C). We confirmed the deletion of *SRR2* in U373MG *SRR2^del^* clone2 and clone5 after transfection with guides and Cas9 (Figure 2B and Figure S1). We then measured the expression of SOX2 in the different cell types. Immunofluorescence showed that most U373MG cells and U373MG *CRISPR* control cells (control) were positive for SOX2, in contrast with U87MG cells, which were used as a negative control (Figure 2C). Notably, U373MG *SRR2^del^* clone2 and clone5 showed a reduction in the number of SOX2-positive cells (Figure 2C,D). In line with this, analysis with Western blots showed a reduction of SOX2 levels in *SRR2^del^* clone2 and clone5 cells (Figure 2E). These results indicate that *SRR2* deletion leads to a reduction in SOX2 expression, and thus demonstrate that SRR2 enhancer activity is required for SOX2 expression in U373MG cells.

### 3.3. SRR2 Deletion Results in Impairment of Cell Proliferation and Stem Cell Activity in Glioma Cells

**Figure 3:**
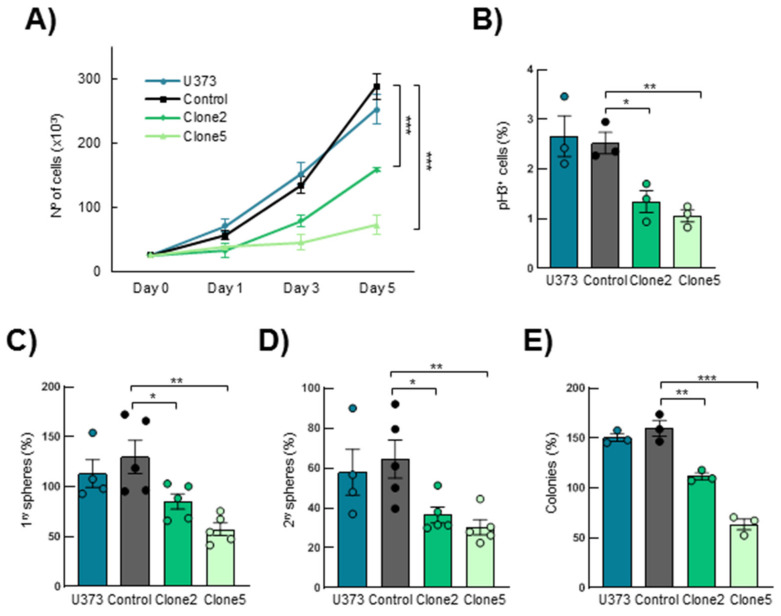
*SRR2* deletion reduces cell proliferation, self-renewal and colony formation capacity. (**A**) Cell growth curve performed in U373MG parental (U373), control, *SRR2^del^* clone2 and clone5 cells at indicated time points (*n* = 3). (**B**) Quantification of positive phospho-Histone H3 (pH3^+^) cells in parental, control, clone2 and clone5 cells (*n* = 3). (**C**) Quantification of primary oncospheres derived from parental, control, clone2 and clone5 cells when plating the same number of U373MG cells (*n* = 5). (**D**) Number of secondary oncospheres obtained after disaggregation of primary oncospheres and plating the same number of cells (*n* = 5). (**E**) Quantification of colony formation from parental, control, clone2 and clone5 cells after 10 days in culture (*n* = 3). The statistical significance was obtained with the Student’s *t*-test (* *p* < 0.05,** *p* < 0.01 and *** *p* < 0.001).

Next, we evaluated the effect of *SRR2* deletion on the tumorigenic capacity of U373MG cells in vitro. By cell counting, we observed less cell growth in U373MG *SRR2^del^* clone2 and clone5 cells compared to *CRISPR* control cells (control) and U373MG parental cells (U373) (Figure 3A). Furthermore, *SRR2*-deleted cells presented a statistically significantly lower number of proliferative, phospho-Histone H3 (pH3)-positive cells (Figure 3B). Indeed, clone2 and clone5 displayed 1.35 and 1.06 positive cells, respectively, compared to 2.53 in control cells and 2.67 in U373 cells. To investigate the role of *SRR2* in stem cell activity, U373, control, clone2 and clone5 cells were seeded in a selective medium for enrichment of the stem cell population. We observed a significant reduction in primary and secondary oncosphere formation efficiency in *SRR2*-deleted GBM cells compared to control and U373 cells, suggesting reduced stem cell activity (Figure 3C,D). In line with this, a reduced colony formation capacity was observed in cells deleted for *SRR2* (Figure 3E). No significant differences were observed between U373 and control cells, which acted similarly in all tests. Moreover, the effect in clone5 was more intense than in clone2 in all experiments. These results confirm that *SRR2* regulates SOX2-driven malignant properties in GBM cells.

### 3.4. SOX2 Overexpression Rescues SRR2 Deletion Effects in U373MG Cells

**Figure 4:**
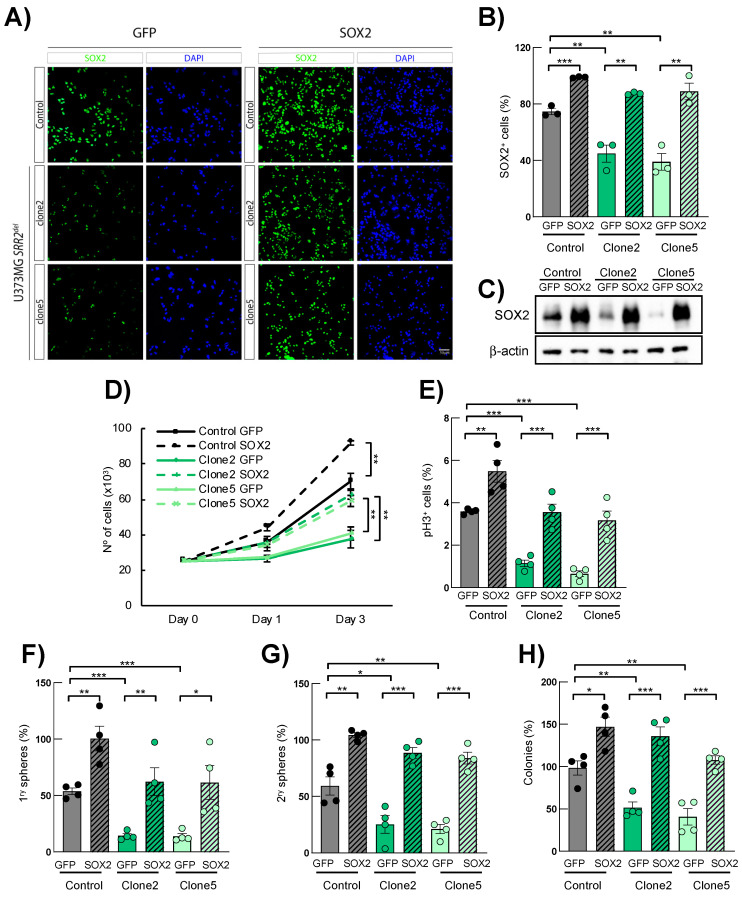
SOX2 overexpression rescues SOX2 expression and malignant properties in *SRR2*-deleted cells. (**A**) Representative images of SOX2 immunofluorescence (scale bar = 50 μm) and (**B**) quantification of SOX2-positive cells in U373MG control, *SRR2^del^* clone2 and clone5 cells infected with control (GFP) and *SOX2* (SOX2) constructs (*n* = 4). (**C**) Representative immunoblot of SOX2 in GFP control, clone2 and clone5 cells in GFP and SOX2 cells (*n* = 3). (**D**) Cell growth curve performed in GFP and SOX2 cells of indicated genotypes and time points (*n* = 4). (**E**) Quantification of positive pH3^+^ cells in GFP- and *SOX2*-overexpressing cells (*n* = 4). (**F**,**G**) Quantification of primary and secondary oncospheres in GFP- and *SOX2*-overexpressing cells (*n* = 4). (**H**) Quantification of colony in indicated genotypes after 10 days in culture (*n* = 4). The statistical significance was obtained with the Student’s *t*-test (* *p* < 0.05, ** *p* < 0.01 and *** *p* < 0.001).

To further determine whether deletion of *SRR2* is necessary for SOX2 expression and oncogenic activity, we next tested whether SOX2 re-activation could restore the impaired phenotypes described in SRR2^del^ cells. Immunofluorescence and Western blotting showed a significant increase in the number of SOX2-positive cells and expression levels in SOX2 control cells, as well as restoration of SOX2 in clone2 and clone5 cells infected with ectopic SOX2 (Figure 4A–C). As expected and previously described [15], forced expression of SOX2 significantly increased proliferation and stem cell properties in control U373MG cells (Figure 4D–H). Interestingly, clone2 and clone5 cells with ectopic expression of *SOX2* also showed higher cell growth and a larger number of pH3+ cells compared to their respective controls (Figure 4D,E). Similarly, the ability to form primary and secondary oncospheres was increased in clone2 and clone5 cells with SOX2 restoration, reaching the numbers of control cells (Figure 4F,G) In agreement with this, clone2 and clone5 cells with SOX2 restoration displayed larger and bigger spheres (Figure S2), as well as increased capability to form colonies at low density compared to their respective controls (Figure 4H). These results show that ectopic expression of *SOX2* rescues several phenotypes of *SRR2^del^* cells, indicating that the expression and the oncogenic activity of SOX2 is, at least in part, mediated by the SRR2 regulatory region.

### 3.5. Expression of p27^KIP1^ and p21^CIP1^ Is Altered in SRR2-Deleted Cells

**Figure 5:**
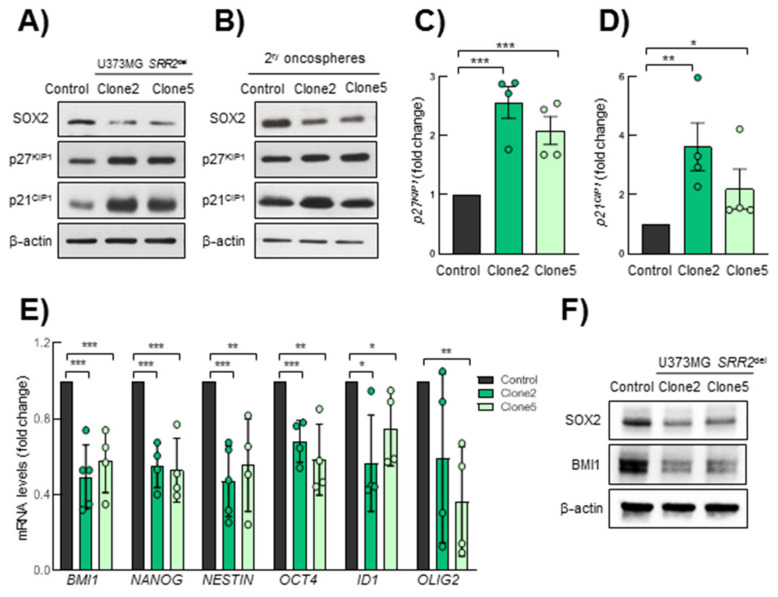
*SRR2*-deleted cells show higher p21^CIP1^ and p27^KIP1^ levels. (**A**) Representative immunoblots of SOX2, p27^KIP1^ and p21^CIP1^ in U373MG control, *SRR2^del^* clone2 and clone5 cells. (**B**) Representative immunoblots of SOX2, p27^KIP1^ and p21^CIP1^ in secondary oncospheres derived from control, clone2 and clone5 cultured in stem cell conditions, References should be cited in numerical order. Please just confirm that all refs are correctly cited, then we will rearrange the order after proofreading. (**C**,**D**) mRNA expression levels of *p27^KIP^* and *p21^CIP1^* by RT-qPCR in clone2 and clone5 referring to control (*n* = 4). (**E**) mRNA expression levels of *BMI1, NANOG, NESTIN, OCT4, ID1* and *OLIG2* by RT-qPCR in clone2 and clone5 referring to control (*n* = 4). (**F**) Representative immunoblots of SOX2 and BMI1 in control, clone2 and clone5 cells. The statistical significance was assessed with the Student’s *t*-test (* *p* < 0.05, ** *p* < 0.01 and *** *p* < 0.001).

p27^KIP1^ and p21^CIP1^, regulating quiescence and acting as cell cycle brakes, both repress *SOX2* expression through *SRR2*, consequently leading to the reduction of proliferation and/or to differentiation programs in the adult brain [20]. At the same time, SOX2 controls the expression of both proteins in different cell types, forming a molecular loop [21,27,28,29]. To study the effect of the *SRR2* deletion on those two proteins, we performed Western blots of p21^CIP1^ and p27^KIP1^ in U373MG control and *SRR2^del^* cells cultured in normal medium (Figure 5A) and as 2^ry^ oncospheres (Figure 5B). We observed that *SRR2^del^* clone2 and clone5 cells presented higher levels of p27^KIP1^ and p21^CIP1^ than control cells in normal conditions (Figure 5A). Furthermore, the protein expression level of p27^KIP1^ and p21^CIP1^ in oncospheres was also higher than in controls (Figure 5B). Moreover, *SRR2*-deleted cells presented increased *p21^CIP1^* and *p27^KIP1^* mRNA expression compared to control cells (Figure 5C,D). Additionally, *SRR2^del^* clone5 cells also showed higher protein levels of p53, the upstream target of p21^CIP1^ (Figure S3). On the contrary, *SRR2^del^* cells presented decreased mRNA and protein levels of well-known stem cell markers such as *BMI1*, *NANOG*, *NESTIN*, *OCT4*, *ID1* and *OLIG2* (Figure 5E,F), supporting the idea that *SRR2* is necessary for the SOX2 regulation of stemness pathways.

### 3.6. SRR2 Deletion Reduces Tumor Initiation and Progression

**Figure 6:**
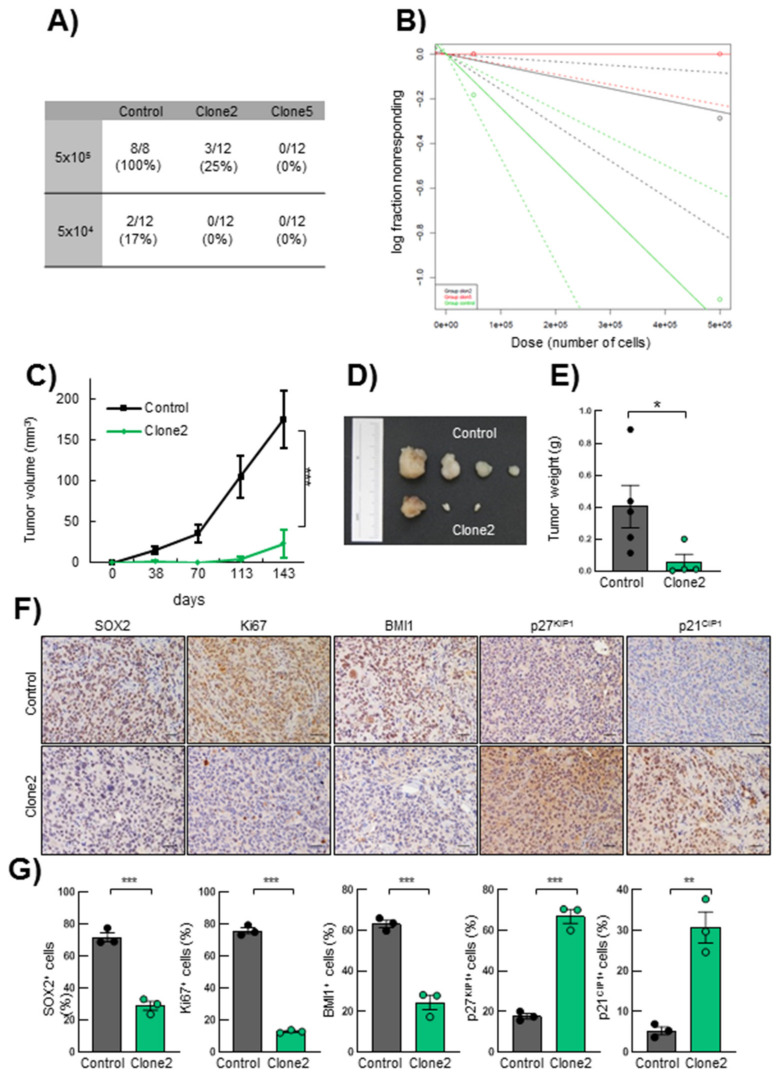
*SRR2* deletion affects tumor initiation and progression in vivo. (**A**) Table of incidence of the number of tumors formed by control, clone2 and clone5 cells after subcutaneous injection of 5 × 10^5^ and 5 × 10^4^ cited cells in 8-week-old immunosuppressed Foxn1^nu^/Foxn1^nu^ nude mice (*n* = 12). (**B**) Logarithmic representation of the tumor initiation frequency after subcutaneous injection of different concentrations of control, clone2 and clone5 cells in nude mice. The incidence of tumor initiation was calculated using the ELDA platform and chi-square tests (χ^2^) (*p* = 5.92 × 10^−5^). (**C**) Graph representing tumor volume developed after subcutaneous injection of control, clone2 and clone5 cells in nude mice measured at the indicated time points (*n* = 12). (**D**) Representative image of tumors derived from control and clone2 cells. (**E**) Average weight of the tumors generated. (**F**–**G**) Representative images and quantification of SOX2, Ki67, BMI1, nuclear p27^KIP1^ and p21^CIP1^ immunohistochemical staining on sections from control and clone2 tumors, scale bar = 100 µm. The statistical significance was obtained with the Student’s *t*-test (* *p* < 0.05, ** *p* < 0.01 and *** *p* < 0.001).

Finally, in order to assess the effect of *SRR2* deletion on the tumorigenic capacity of glioma cells in vivo, we performed a tumor initiation assay in immunodeficient mice by injecting different concentrations of U373MG control, *SRR2^del^* clone2 and clone5 cells. One hundred percent of animals injected with 100,000 control cells developed tumors versus 25% in those injected with the same numbers of clone2 cells, and 0% in those receiving clone5 cells (Figure 6A). Using the ELDA tool, we calculated the frequency of tumor-initiating cells. This revealed a significantly lower frequency in clone2 and clone5 cells, respectively, of 1/1.9.10^6^ cells and no tumor-initiating cells (1/∞), compared to 1/4.10^5^ in control cells (Figure 6B). Of note, clone5 cells were unable to form tumors, further showing a stronger effect than clone2. Moreover, tumors derived from *SRR2*-deleted clone2 cells were significantly smaller compared to controls (Figure 6C). Indeed, subcutaneous tumors derived from clone2 cells reached a volume of less than 25 mm^3^ after 140 days, compared to tumors from control cells that grew to an average of 170 mm^3^ during the same time (Figure 6C,D). In line with these results, the weight of clone2 cell-derived tumors were lower than tumors from control cells (Figure 6E). We then performed immunohistochemistry with SOX2, Ki67, BMI1, p27^KIP1^ and p21^CIP1^. Tumors generated from clone2 displayed lower expression of SOX2, Ki67 and BMI1 compared to the tumors formed by control cells (Figure 6F,G), validating the in vitro observations of impaired proliferation and stemness of *SRR2^del^* cells. In contrast, clone2-derived tumors exhibited higher levels of p27^KIP1^ and p21^CIP1^ (Figure 6F,G). In summary, these results reveal that *SRR2* is required for SOX2 tumorigenic activity.

## 4. Discussion

SOX2 expression involves a complex network of transcriptional, post-transcriptional and post-translational regulators. The downstream *SOX2 regulatory region 2* (*SRR2*) exerts relevant control on SOX2 expression. In this regard, *SRR2* is a region involved in the expression of SOX2 in pluripotent stem cells during development and in the maintenance of stem characteristics in various adult stem cell populations [16]. In this study, we dissected the influence of *SRR2* on SOX2 expression in glioma cells and demonstrated that the deletion of this regulatory region significantly reduces SOX2 levels, and hence activity in these cancer cells.

Our results confirm that *SRR2* mediates repression of *SOX2* expression in differentiation conditions in GSCs and NSCs. This effect might be mediated via different mechanisms. We reveal that it happens through p27^KIP^ binding. p21^CIP1^ has also been identified as a regulator of SOX2 expression in NSCs via binding on SRR2 [21]. Additionally, OCT3/4 and SOX2 complexes can bind to *SRR2* and promote SOX2 expression [30]. Our results extend the understanding and the relevance of p27^KIP1^ repressive action for modulation of *SOX2* levels because conclusions in previous studies were limited to physiological conditions. In this regard, we previously showed that p27^KIP1^ recruits a repressor complex on *SRR2* to repress *SOX2* expression during embryonic stem cell differentiation [20].

We prove that *SRR2* deletion leads to a reduction in the expression of *SOX2* in glioma cells. Previous studies showed that SOX2 expression depends on the epigenetic status of *SRR2* in NSCs [31]. Indeed, increasing CpG methylation in *SRR2* coincides with lower expression of *SOX2* and differentiation into neurons [31]. *SRR2* contains a functional SOX-binding site whose mutations result in complete loss of transcriptional stimulating activity, demonstrating the positive feedback of SOX2 on its own expression [19]. Our results highlight that SRR2 controls SOX2 expression in brain stem cells not only under physiological but also under pathological conditions. Indeed, our findings experimentally demonstrate the relevance of *SRR2* as an enhancer of SOX2 expression in cancer cells because deletion of *SRR2* in GBM cells leads to a reduction of SOX2 expression.

The de-repression of SOX2 has been associated with a wide range of cancer types, including GBM [3]. In the latter, high levels of SOX2 are associated with GSCs and lower patient survival and its activity is required for tumor maintenance and progression, because silencing of the gene in GBM cells reduces the capacity to proliferate, migrate and self-renew, whereas *SOX2* overexpression promotes an increase in cell proliferation, migration and self-renewal capacity [3,10,11,12,13,14,15]. Here, we report that deletion of *SRR2* in glioma cells leads to a reduction in proliferation and stem cell activity in vitro. Notably, in vivo, our xenograft models revealed that the lack of *SRR2* results in an impairment of tumor initiation and growth. Furthermore, ectopic *SOX2* overexpression in *SRR2* enhancer-deleted cells restored SOX2 expression as well as proliferation and stemness capacities. These results reveal that the *SRR2* regulatory region is required for SOX2 expression and hence tumorigenic activity in cancer cells.

To further characterize proteins involved in the regulation of SOX2 expression, we studied p27^KIP1^ and p21^CIP1^. We analyzed these proteins because (i) SOX2’s proliferative function in cancer cells is exerted, at least in part, through direct repression of p21^CIP1^ and p27^KIP1^ expression [27,28]; (ii) there is an interaction between SOX2 and p27^KIP1^ in mice, in which we have just demonstrated that *Srr2* plays a role [20,29]. We observed an increased expression of both p21^CIP1^ and p27^KIP1^ in *SRR2*-deleted glioma cells. Together with the information presented above, these results indicate the existence of repressive, cross-regulatory interactions between SOX2, p21^CIP1^ and p27^KIP1^ that play important roles in cancer cells. In summary, our results highlight the central role of *SRR2* to maintain high levels of SOX2 expression, and hence malignant properties of glioma cells establishing the requirement for this regulatory region for SOX2-driven tumorigenic activities.

## 5. Conclusions

*SRR2* deletion by CRISPR/Cas9 technology leads to a reduction in *SOX2* expression in glioma cells. Consequently, cell growth and proliferation are reduced in these cells, while self-renewal capacity is impaired in GSCs. This is associated with an increase in the expression of the negative regulators of the cell cycle, p21^CIP1^ and p27^KIP1^. Furthermore, deletion of *SRR2* impairs tumor initiation and progression capacity of cancer cells in vivo. Our results indicate that prevention of *SRR2* activity leads to a reduction of oncogenic properties promoted by SOX2, suggesting that *SRR2* may represent a novel therapeutic target in the treatment of GBM, to tackle GSCs, and hence relapse of these lethal tumors.

## Data Availability

The data presented in this study are available on request from the corresponding author.

## Supplementary Materials

The following are available online at https://www.mdpi.com/article/10.3390/cancers13071574/s1, Figure S1. Clone2 and Clone5 sequenced and matching in genomic human sequence of *SRR2*. Figure S2. Ectopic expression of *SOX2* rescues stem cell activity in *SRR2* deleted cells. Figure S3. A) Expression of p53 in *SRR2* deleted GBM cells. Figure S4. Uncropped Western Blots. Table S1. Primers for CRISPR genome editing. Table S2. Primers used for Genotyping and ChIP assay.
