# Supplementary material for: CRISPR/Cas9 Deletion of SOX2 Regulatory Region 2 (SRR2) Decreases SOX2 Malignant Activity in Glioblastoma
Source: Cancers (Basel). 2021 Mar 29;13(7):1574. doi: 10.3390/cancers13071574 (PMC8037847; doi:10.3390/cancers13071574)
Supplement: Supplementary file 1 [file cancers-13-01574-s001.pdf]

Supplementary Materials: *CRISPR/Cas9* Deletion of *SOX2 Regulatory Region 2* (*SRR2*) Decreases SOX2 Malignant Activity in Glioblastoma

Ander Saenz-Antoñanzas, Veronica Moncho-Amor, Jaione Auzmendi-Iriarte, Alejandro Elua-Pinin, Karine Rizzoti, Robin Lovell-Badge and Ander Matheu

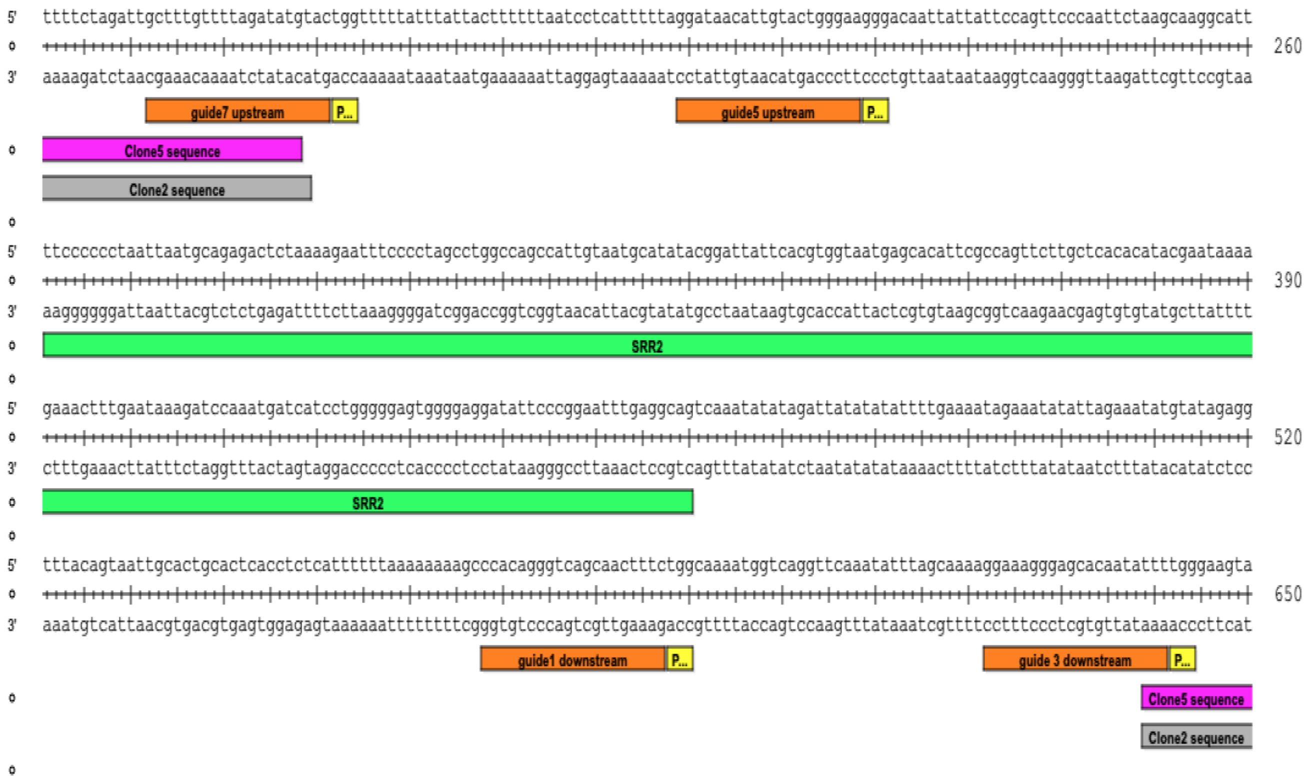

Figure S1. Clone2 and Clone5 sequenced and matching in genomic human sequence of *SRR2*. *SRR2* (200bp) is marked by a green box. The location of the external (guide 7_5′ and guide 3_3′) and internal (guide 5_5′ and guide 1_3′) sgRNAs (orange box) is labelled including their PAM sequence (in yellow). Sequence of the Clone2 (grey box) and Clone5 (magenta box) cell lines carrying *SRR2* deletion represented from sequencing. The breakpoint is shown above the colourful squares that indicate the nucleotides remained from each clone sequence following the deletion.

A)

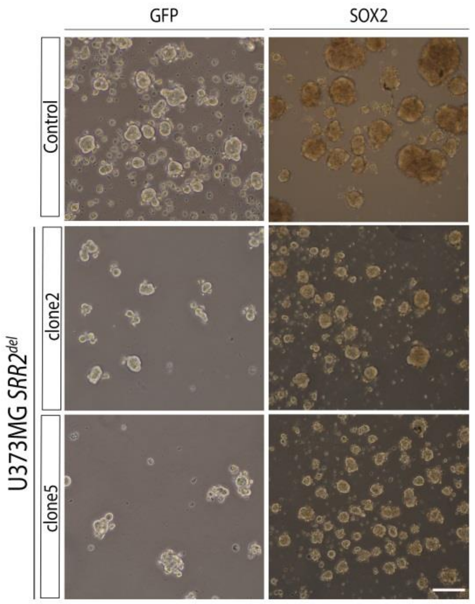

B)

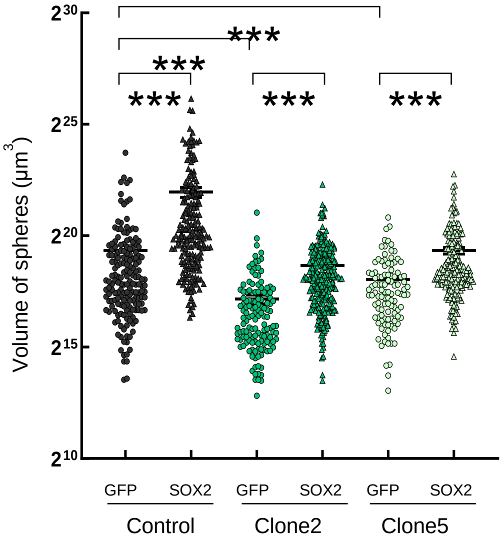

Figure S2. Ectopic expression of *SOX2* rescues stem cell activity in *SRR2* deleted cells. A) Representative images (scale bar=200μm) and **B)** volume of primary oncospheres derived from control, clone2 and clone5 cells with ectopic expression of *SOX2* compared to GFP control, clone2 and clone5 cells and secondary oncospheres obtained after disaggregation of primary oncospheres. (n=4).

A

B

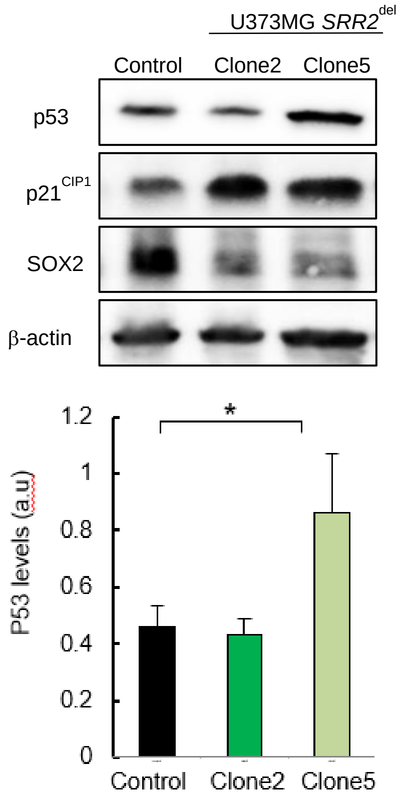

Figure S3. A) Expression of p53 in *SRR2* deleted GBM cells. Representative immunoblots of p53, $p21^{CIP1}$ and SOX2 in U373MG control and U373MG $SRR2^{del}$ clone2 and clone5 cells. **B)** Quantification of p53 expression in GBM cells (n=3).

Figure 1

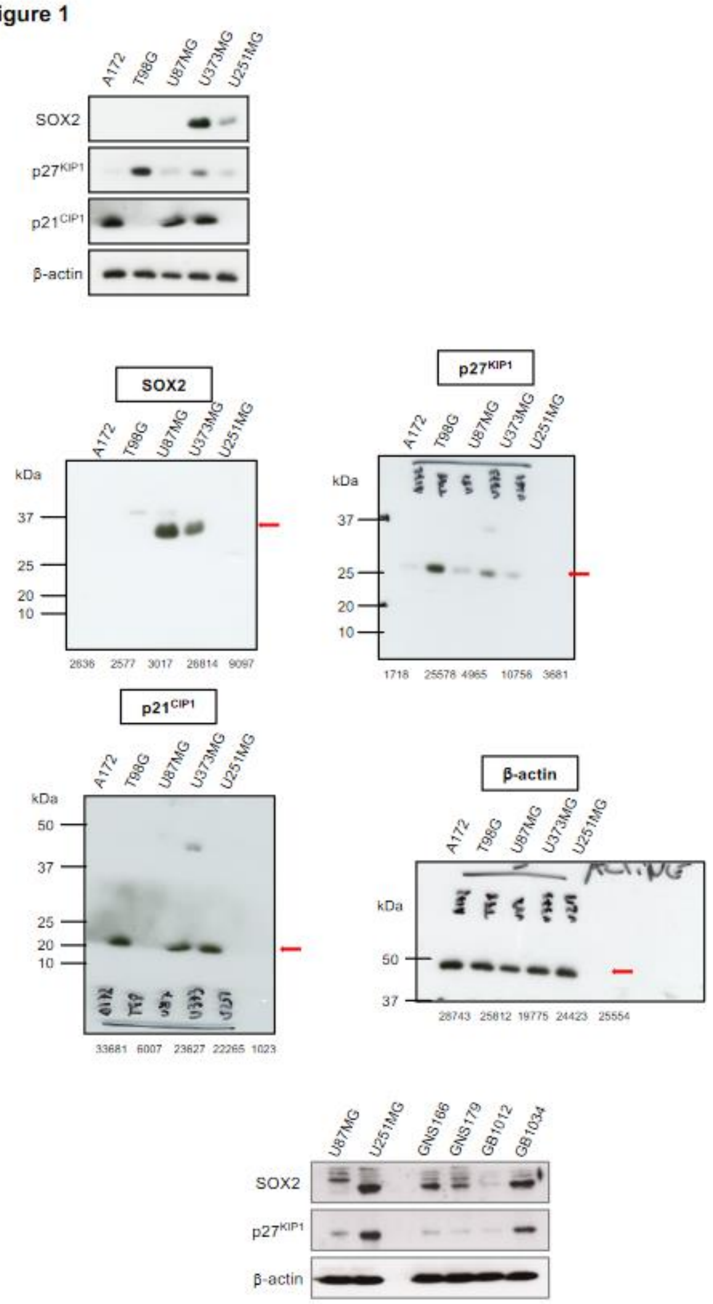
A172
T98G
U87MG
U373MG
U251MG
SOX2
p27KIP1
p21CIP1
β-actin
kDa
37
25
20
10
50
2636 2577 3017 26814 9097
1718 25578 4965 10756 3681
33681 6007 23627 22265 1023
28743 25812 19775 24423 25554
GNS166
GNS179
GB1012
GB1034

Figure 2

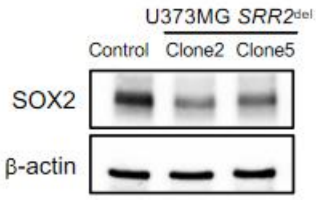
U373MG SRR2del
Control
Clone2
Clone5
SOX2
β-actin

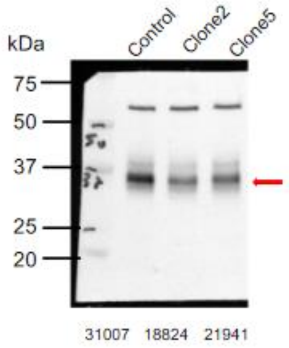
SOX2
kDa
Control
Clone2
Clone5
75
50
37
25
20
31007
18824
21941

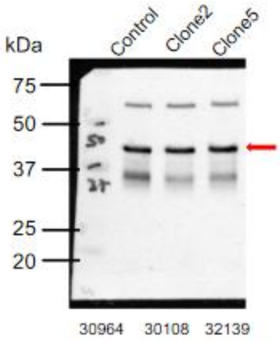
β-actin
kDa
Control
Clone2
Clone5
75
50
37
25
20
30964
30108
32139

Figure 4

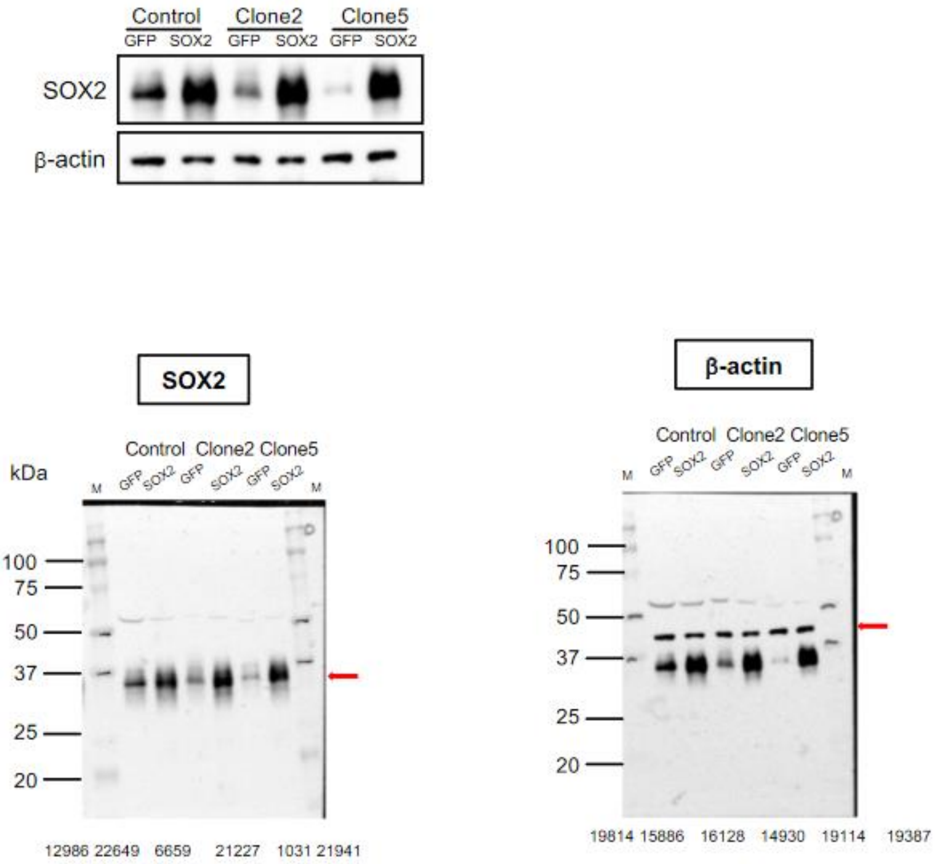

Control
Clone2
Clone5
GFP SOX2 GFP SOX2 GFP SOX2
SOX2
β-actin
SOX2
kDa
M
100
75
50
37
25
20
12986 22649 6659 21227 1031 21941
β-actin
19814 15886 16128 14930 19114 19387

Figure 5

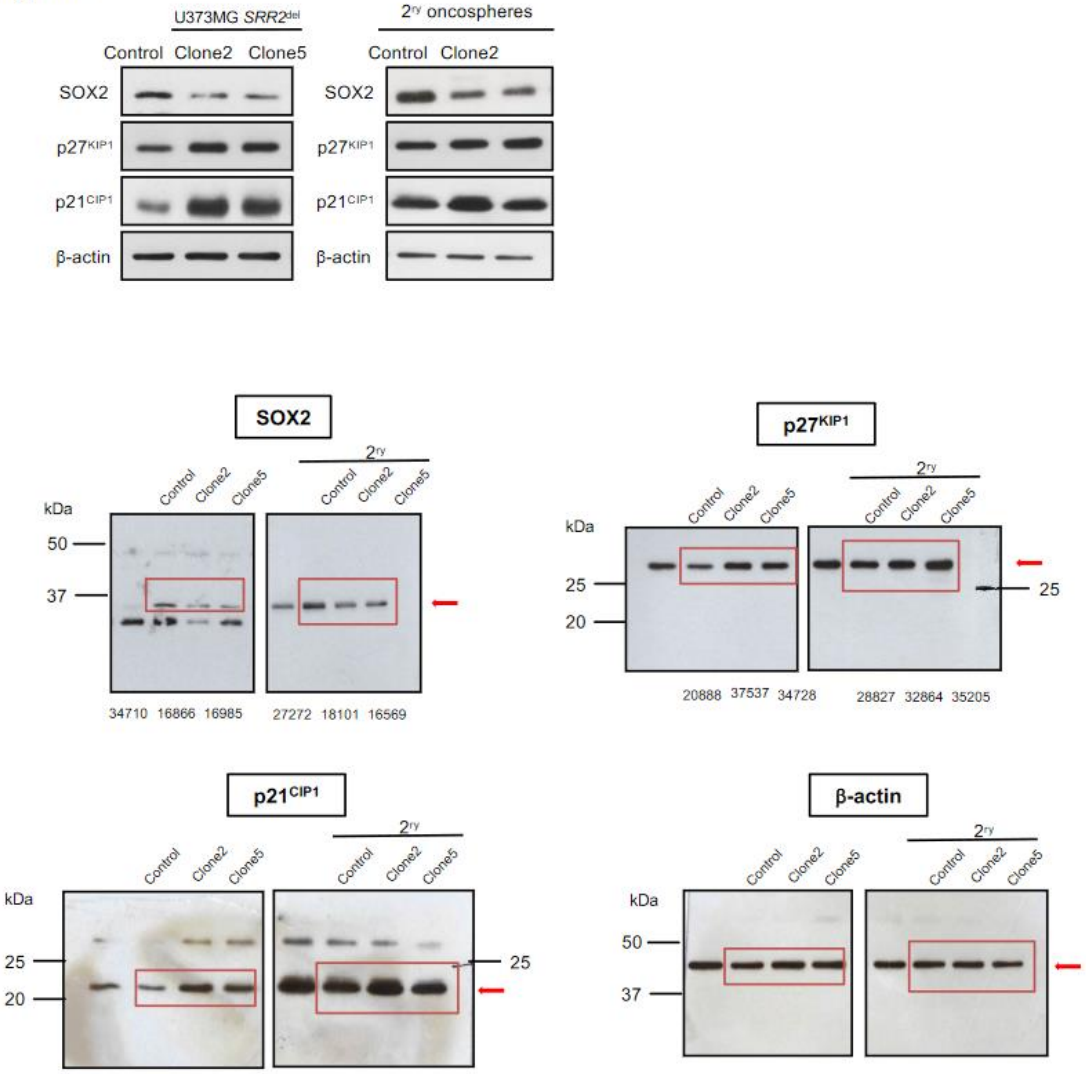
U373MG SRR2del
2ry oncospheres
Control Clone2 Clone5
Control Clone2
SOX2
p27KIP1
p21CIP1
β-actin
SOX2
p27KIP1
p21CIP1
β-actin
kDa
50
37
25
20
34710 16866 16985
27272 18101 16569
20888 37537 34728
28827 32864 35205

Figure 5

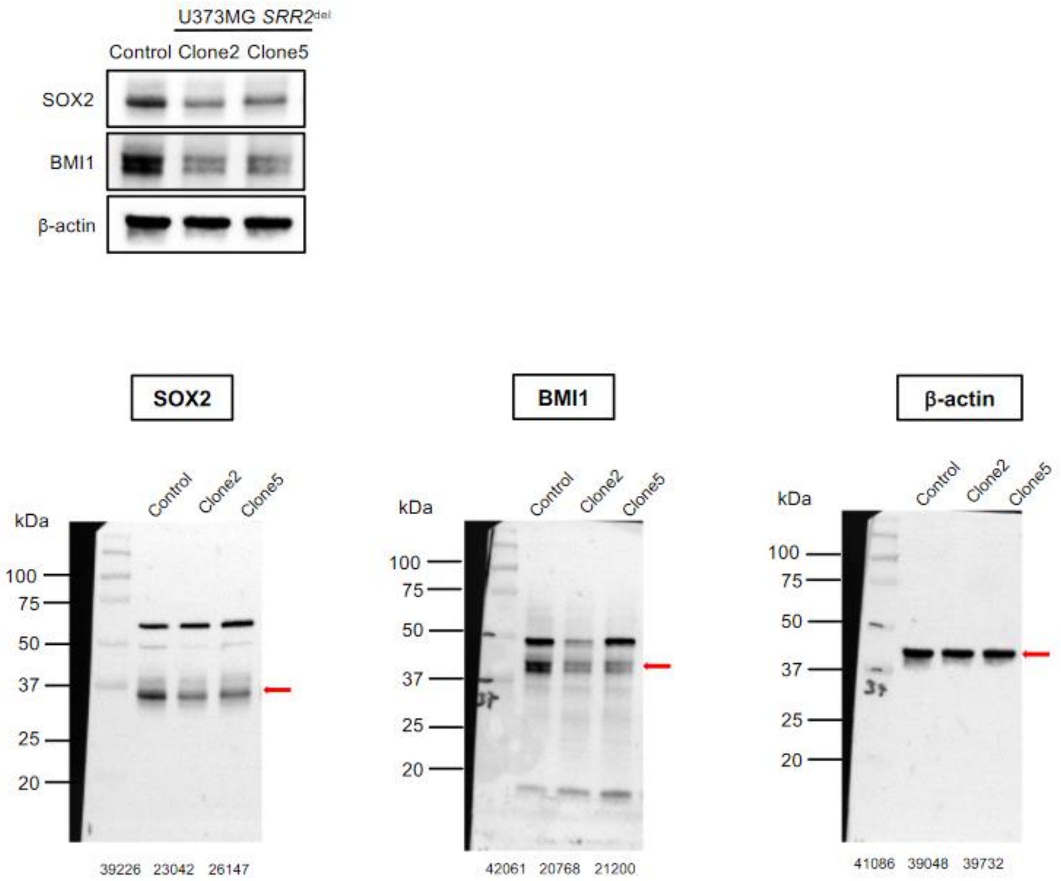
U373MG SRR2del
Control Clone2 Clone5
SOX2
BMI1
β-actin
SOX2
BMI1
β-actin
kDa
100
75
50
37
25
20
Control
Clone2
Clone5
39226 23042 26147
42061 20768 21200
41086 39048 39732

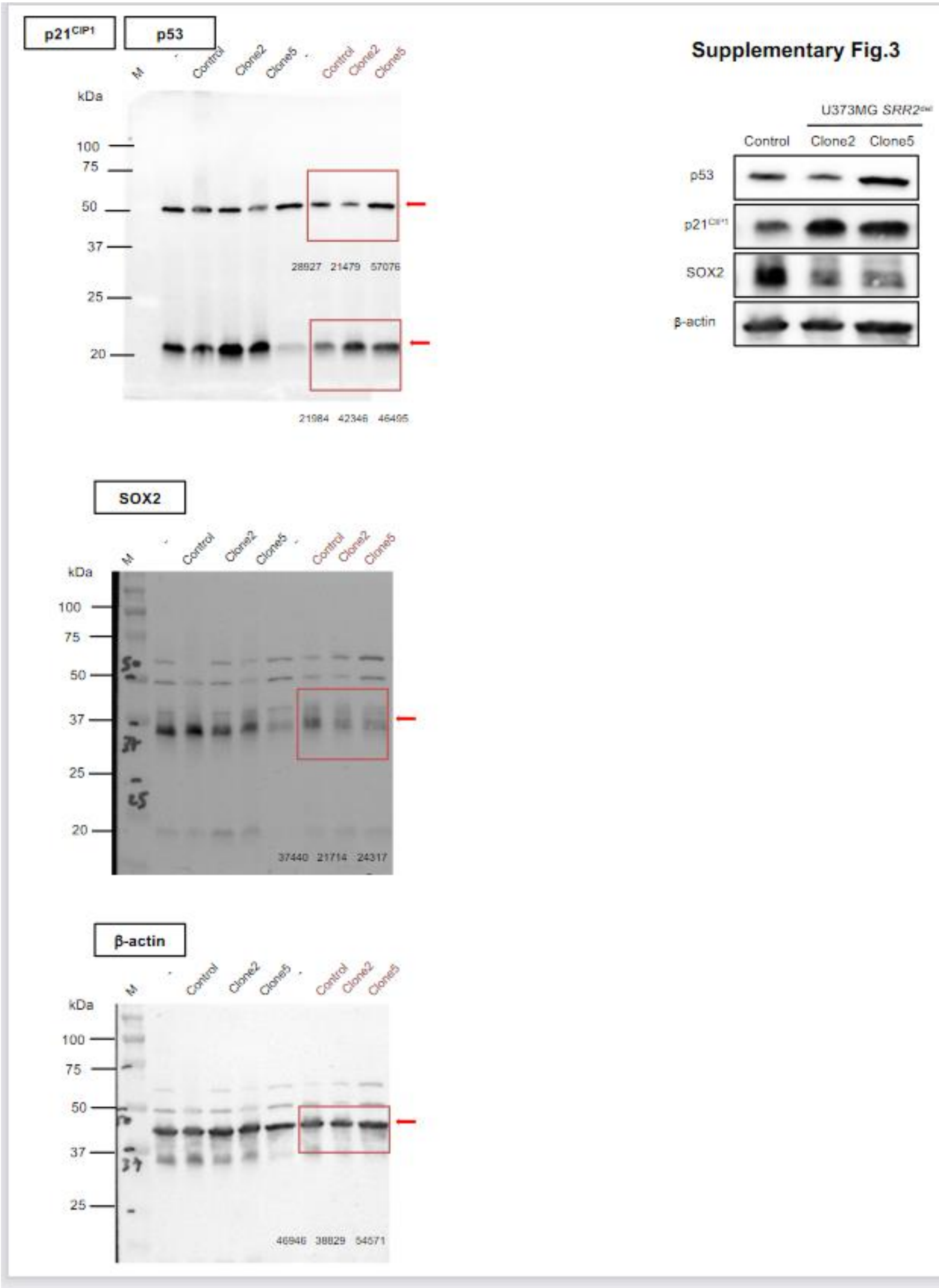

Figure S4. Uncropped Western Blots.

Table S1. Primers for CRISPR genome editing

sgRNA	*Primer name*	*Sequence 5' to 3' (20-mer sgRNA recognition sequences in bold)*
**GUIDE 7 UPSTREAM SRR2**	G7 UP SSR2_F	5' CACC **GCT TTG TTT TAG ATA TGT AC** 3'
	G7 UP SSR2_R	5' AAACGTACATATCTAAAACAAAGC 3'
**GUIDE 5 UPSTREAM SRR2**	G5 UP SRR2_F:	5' CACC **GGA TAA CAT TGT ACT GGG AA** 3'
	G5 UP SRR2_R:	5' AAACTTCCCAGTACAATGTTATCC 3'
**GUIDE 1 DOWNSTREAM SRR2**	G1 DOWN SSR2_F	5' CACCG **CCA CAG GGT CAG CAA CTT TC** 3'
	G1 DOWN SSR2_R	5' AAACGAAAGTTGCTGACCCTGTGGC 3'
**GUIDE 3 DOWNSTREAM SRR2**	G3 DOWN SRR2_F:	5' CACC **GGA AAG GGA GCA CAA TAT TT** 3'
	G3 DOWN SRR2_R:	5' AAAC AAATATTGTGCTCCCTTTCC3'
**pX330 plasmid**	uniR	AAAAGCACCGACTCGGTGCC

Table S2. Primers used for Genotyping and ChIP assay		
Primer name	Sequence 5' to 3'	Application
Sox2-SRR2-F	5’ ATTTATTCAGTTCCCAGTCCAAGC 3’	qPCR after ChIP
Sox2-SRR2-R	5’ CCCTCTCCCCCCACGC 3’	qPCR after ChIP
SRR2_FORWARD	5’ GCCACTAAGTTAGCTCATCC 3’	Genotyping
SSR2_REVERSE	5’ ATATGAAGGGGTAGAAGGGG 3’	Genotyping
